# Transdermal Immunization with Microparticulate RSV-F Virus-like Particles Elicits Robust Immunity

**DOI:** 10.3390/vaccines10040584

**Published:** 2022-04-10

**Authors:** Sucheta D’Sa, Kimberly Braz Gomes, Grace Lovia Allotey-Babington, Cemil Boyoglu, Sang-Moo Kang, Martin J. D’Souza

**Affiliations:** 1 Center for Drug Delivery Research, Vaccine Nanotechnology Laboratory, Mercer University, Atlanta, GA 30341, USA; sucheta.dsa@gmail.com (S.D.); kimbrazgomes@gmail.com (K.B.G.); glallotey-babington@ug.edu.gh (G.L.A.-B.); cboyoglu@yahoo.com (C.B.); 2 Center for Inflammation, Immunity & Infection, Institute for Biomedical Sciences, Georgia State University, Atlanta, GA 30303, USA; skang24@gsu.edu

**Keywords:** RSV, virus-like particles (VLPs), transdermal, microparticles

## Abstract

No approved vaccines against respiratory syncytial virus (RSV) infections exist to date, due to challenges arising during vaccine development. There is an unmet need to explore novel approaches and a universal strategy to prevent RSV infections. Previous studies have proven the immune efficacy of virus-like particles (VLPs) consisting of RSV fusion (F) protein, yielding a highly immunogenic RSV-F VLP subunit vaccine. In this study, RSV-F VLP (with or without MPL^®^) was added to a polymer mix and spray-dried, forming microparticles. The formulations were transdermally administered in C57BL/6 mice to evaluate vaccine efficacy. The transdermal delivery of RSV-F VLP + MPL^®^ was more effective in clearing lung viral loads and preventing weight loss after RSV challenge. At the cellular level, MPL^®^ augmented the vaccine response in microparticulate form, which was evidenced by higher serum and lung antibody titers, and lower lung viral titers in the vaccinated groups. These preliminary results validate the effectiveness of the RSV-F VLP microparticulate vaccine via the transdermal route due to its potential to trigger robust immune responses.

## 1. Introduction

Respiratory syncytial virus (RSV) is one the frequent causes of severe respiratory illness and mortality in children, the aged, and immunocompromised populations [1]. RSV causes upper and lower respiratory tract infections (LRTIs) such as bronchiolitis and pneumonia. RSV infects a likely 64 million people in the world, contributing to a death toll of 160,000 each year [2]. Globally, RSV is estimated to cause 33 million new episodes of LRTI in children under the age of 5, causing nearly 3.2 million hospitalizations and ~118,000 deaths each year [1,2]. In adults, RSV infections become more severe with age, where ~5% of older patients are infected with the disease annually. In the United States alone, an estimated 177,000 hospitalizations and 14,000 deaths each year are recorded among adults [2,3]. Effective therapies to combat the virus are not widely available, and hence, in the past few decades, a significant amount of time has been spent in developing a promising strategy to prevent the spread of RSV infections using subunit vaccines, live-attenuated or inactivated viruses, and live vector vaccines. Despite several vaccine candidates entering clinical trials, no licensed RSV vaccine is available to date. This is partially due to an unfortunate outcome following a clinical trial in the 1960s, where children were administered formalin-inactivated RSV vaccine (FI-RSV) in combination with alum. Several children who were vaccinated with FI-RSV developed vaccine-enhanced respiratory disease, resulting in a surge in hospitalization rates and death in two cases [1]. It was revealed that FI-RSV vaccine-enhanced respiratory disease is mainly due to the activation of abnormal Th2 responses [1].

RSV is a single-stranded RNA-enveloped virus, belonging to the Paramyxoviridae family, with three major transmembrane proteins—the (G) attachment glycoprotein, the fusion (F) glycoprotein, and the small hydrophobic (SH) protein [4]. The G protein contributes to viral attachment, and the F protein is essential for entry into the host cell, making them target proteins for exploration as potential vaccine candidates [4]. Such viral proteins presented as virus-like particles (VLPs) or recombinant antigens are highly immunogenic and known to induce protection [5]. VLP-based vaccines can induce the innate and adaptive immune systems, with licensed vaccines available against the human papilloma and hepatitis B viruses. VLPs are genetically engineered molecules structurally composed of multiple repeats of antigenic proteins, which resemble the viral structure but are non-infectious and non-replicating due to a lack of genetic material. In the case of RSV, the fusion (F) and attachment glycoprotein (G) induce neutralizing antibodies and display various T-cell epitopes [6]. Self-assembly of the antigenic F protein into VLPs has previously been shown to induce robust RSV-specific immune responses [5,7,8]. For this study, VLPs consisting of the RSV-F protein were prepared using the previously established insect cell expression system [5].

VLPs comprising RSV-F and/or G glycoproteins have been previously shown to activate antigen-specific antibody responses capable of defending against RSV infection in murine models [9,10]. To further enhance immunogenic responses, previous research has shown that encapsulating antigens, particularly VLPs, within a micro- or nanoparticulate delivery system can produce robust immune responses [11]. In addition, encapsulated antigens are often presented as pathogens or foreign substances to the immune system and can be taken up better by antigen-presenting cells (APCs) which activate downstream signaling pathways of the innate and adaptive immune system.

The effectiveness of many vaccines is often based on the time during which the immune system is exposed to an antigen, an inherent characteristic that can be enhanced with the addition of molecules known as adjuvants. Adjuvants are substances known to potentiate the magnitude and duration of a specific immune response to an antigen. Most vaccines approved for use employ adjuvants, since protection against the antigen may be achieved for longer periods by reducing the antigen dose or the frequency of immunizations required to trigger an immune response [12]. A commonly used adjuvant, known as Monophosphoryl lipid A (MPL^®^), a Toll-like receptor 4 (TLR-4) agonist derived from endotoxin and an efficient stimulator of cellular and humoral responses, is the first and only TLR ligand to be used in licensed human vaccines, [13] and hence was used as an adjuvant in this study.

Conventionally, intramuscular (I.M.) injections have been the choice for vaccine administration, owing to long-term efficacy and safety. However, this route is not favorable as many individuals exhibit a fear of needles. Researchers have invested time and effort in exploring alternate routes of administration for vaccines and medications, with special attention to needle-free vaccinations [14]. In recent years, the transdermal route has gained popularity due to its advantages related to patient compliance, ease of administration, lower doses, and especially because of the ability to benefit from the immunocompetence of the skin [14]. In transdermal vaccination, dendritic cells and Langerhans cells (LCs) residing in the dermis can engulf the antigen, circulate to the primary and secondary lymphoid organs, and interact with naïve T cells, leading to the activation of cellular immunity [15]. Transdermal delivery is considered a promising route of administration due to the presence of skin-associated lymphoid tissue composed of keratinocytes, Langerhans cells, dermal dendritic cells, lymphatic vessels, and subsets of T-lymphocytes [16]. A form of transdermal delivery system using microneedles penetrates the outer stratum corneum of the skin and allows the transport of macromolecules such as vaccine proteins and peptides that otherwise pose a challenge when delivered across the skin passively [17]. Owing to short needle lengths, microneedles do not stimulate the nerve endings in the dermis, and thus are a non-invasive and pain-free form of vaccination [17,18]. Previous studies have shown that microneedle patch vaccination can induce more robust and long-lasting immune responses than conventional I.M. vaccination due to skin dendritic cells [8,11]. Moreover, the non-invasive delivery of vaccines via a microneedle patch would be greatly acceptable for individuals, especially children with a phobia of needles.

In this study, it was hypothesized that the transdermal delivery of a novel vaccine via the incorporation of a viral antigen such as the RSV fusion (F) protein VLPs in a polymeric matrix may not only result in enhanced uptake by immune cells, but also provide improved antigen presentation and recognition by the immune system. To demonstrate this, our proof-of-concept study focused on the development of a spray-dried RSV-F VLP microparticle vaccine with the addition of MPL^®^ as an adjuvant, by utilizing a C57BL/6 murine model to investigate immune responses and protection against RSV.

## 2. Materials and Methods

### 2.1. Materials

FI-RSV, RSV-F VLP, and mouse anti-RSV F monoclonal antibodies were provided as a gift by Dr. Sang-Moo Kang from Georgia State University. RPMI 1640 medium was purchased from Sigma Aldrich, St. Louis, MO, USA. Fluorescently labeled anti-mouse antibodies, CD4-FITC and CD8a-PE, were purchased from BD Biosciences, Franklin Lakes, NJ, USA. Six- to eight-week-old female C57BL/6 mice (*n* = 5) were purchased from Charles River Laboratories (Wilmington, MA, USA) and maintained in the Mercer University (Atlanta, GA, USA) animal facility under the guidelines of the Mercer University Institutional Animal Care and Use (IACUC) protocol. Hydroxypropyl methylcellulose acetate succinate (HPMCAS, Shin-Etsu AQOAT^®^), 30% (*w/v*) aqueous dispersion of cellulose acetate phthalate (Aquacoat^®^ CPD), and ethyl cellulose (EC), 30% (*w/v*) aqueous dispersion (AQOAT^®^) were gift samples from FMC biopolymers (Philadelphia, PA, USA). Trehalose, glycol chitosan, and Monophosphoryl lipid A (MPL^®^) were purchased from Sigma Aldrich (St. Louis, MO, USA). The AdminPatch^®^ 1200 microneedle array was purchased from nanoBioSciences LLC. All other materials used in the study were of analytical grade.

### 2.2. Preparation of RSV-F VLPs

RSV-F VLPs composed of the RSV A2 fusion (F) protein were prepared by infecting Sf9 insect cells to express recombinant influenza virus M1 and RSV-F proteins, purified as described previously [5,7]. Briefly, centrifugation was carried out to obtain cell culture supernatants at 6000 rpm for 20 min at 4 °C [7]. The VLPs were concentrated and purified through an optimized sucrose gradient at 30,000 rpm for 1 h using an ultracentrifugation step [5]. The VLP bands between specific gradients were collected and then diluted with phosphate-buffered saline (PBS) and pelleted at 28,000 rpm for 40 min at 4 °C [5]. PBS was used to resuspend the VLPs at 4 °C [5].

### 2.3. Preparation and Characterization of RSV-F VLP Microparticulate Vaccine

The RSV-F VLP microparticulate vaccine formulation was prepared using the spray-drying technique. RSV-F VLP was incorporated into a biodegradable, cellulose-based matrix consisting of the following components (*w/w*): 20% CPD, 32% EC, 35% HPMCAS, 4% (*w/w*) trehalose, 4% glycol chitosan, and 5% RSV-F VLP + MPL^®^. HPMCAS was dissolved in deionized water at a pH of 8.0 with overnight stirring, while CPD dispersion was adjusted to pH 6.0 using 1 N NaOH. Aquacoat ECD dispersion was used without any dilution. CPD dispersion was diluted to 5% by adding 4 mL of 30% dispersion to 100 mL of deionized (DI) water, and later adjusted to a pH of 6.0. Similarly, in a separate beaker, 300 mg of HPMCAS was added to 50 mL of DI water and adjusted to a pH of 8.0 to prepare a working stock. For a product yield of 100 mg, a solution comprising a mixture of polymers was prepared (0.1% solid content for spray drying). An antigen: adjuvant ratio consisting of RSV-F VLP: MPL^®^ at a ratio of 1:2 was added to the polymer mixture, followed by 4 mg each of chitosan glycol and trehalose. Finally, 0.01% *v/v* Tween^®^20 was added to the formulation to prevent the sticking of dried product to the heating chamber. The pH of the suspension was adjusted to 7.0 using 1N NaOH to solubilize the suspended polymers, followed by q.s. to final volume with deionized water, and it was subsequently spray-dried using a Buchi B-290 mini spray dryer (Buchi Corporation, New Castle, DE, USA). The solution flowed through a two-fluid nozzle of 0.5 mm diameter maintained at −5 °C. The inlet and outlet temperatures were controlled at 120 °C and 80 °C, respectively. The aspirator was set at 100%, and the flow rate was adjusted to 25 mL/h to obtain the RSV-F VLP vaccine microparticles, which were stored at −20 °C for future use.

### 2.4. Characterization of Vaccine Microparticles

The uptake of the vaccine particles by immune cells was determined by measuring the size of the microparticles. Additionally, zeta potential is essential to predict the microparticle stability and uptake during administration. Therefore, the size and charge of the RSV-F VLP-loaded microparticles were measured using the laser diffraction technique and analyzed using the Malvern Zetasizer^®^ Nano ZS. Briefly, 1 mg of microparticles was weighed and suspended in 2 mL of citrate buffer (pH 3.5). The suspension was transferred to a disposable cuvette and evaluated for its size distribution. The surface morphology of particles was obtained using the Phenom Pure Desktop^®^ scanning electron microscope (SEM). The microparticles were placed on a metal holder with a carbon film, and the excess particles were blown away using a compressed gas duster. The image was taken in SEM mode with 4100×, at an angle of 28.7°, and an accelerating voltage of 20 kV. The loading efficiency was determined using the Micro BCA^™^ protein assay kit by extracting entrapped antigen in PBS.

### 2.5. Transdermal Immunization, Live Challenge, and Blood Collection

**Figure 1:**
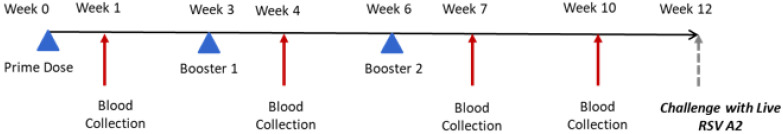
Immunization schedule. A prime dose of 5 µg of RSV-F VLP subunit vaccine was administered to mice on week 0, followed by a booster on weeks 3 and 6. Blood samples were collected and IgG titers were measured on weeks 1, 4, 7, and 10. Live RSV A2 virus challenge was induced intranasally on week 12.

Six to eight-week-old female C57BL/6 mice (a total of 25 mice) were procured from Charles River Laboratories, Inc., (Wilmington, MA, USA). The various groups used for the study are presented in Table 1. For the study design, as shown in Figure 1, a schedule of one prime (week 0) and two booster doses (week 3 and 6) were administered either via the I.M. route, using a syringe (25-gauge needle) or via the transdermal (T.D.) route, using microneedles i.e., the AdminPatch^®^ 1200. The microneedle patch was placed onto the shaved skin of mice and pressure was applied to create pores, following which, the microparticle formulation was applied as a suspension.

For the I.M. group, a dose of 1 µg of FI RSV (equivalent to 1 × 10^6^ plaque-forming units (PFUs) was administered. For transdermal groups, mice were immunized with a suspension or microparticulate (MP) form of RSV-F VLP (5 µg per mouse) with MPL^®^ (10 µg per mouse). The naïve control group was administered 1× PBS solution via the transdermal route. Blood samples were collected at weeks 1, 4, 7, and 10 and quantitatively analyzed for antibody titers using ELISA. Naïve and immunized mice were intranasally challenged with RSV A2 (1 × 10^6^ PFU) live virus under isoflurane vaporization at week 12 to determine vaccine efficacy. Blood samples were collected up to 3 weeks after the prime-boost administration. The body weights of mice were measured every day for 6 days following challenge. The mice were euthanized and individual organs, including the lungs, axillary and inguinal lymph nodes, spleen, and bone marrow, were collected at week 14. The animal experiments were carried out as per approved protocols by Mercer University’s Institutional Animal Care and Use Committee (IACUC).

### 2.6. Antibody Responses in Serum and Lung Using ELISA

RSV-F protein-specific total IgG titers were quantified in serum and lung homogenates using an enzyme-linked immunosorbent assay (ELISA). Blood sampling in mice was carried out prior to vaccination and on weeks 1, 4, 7, and 10. Serum was obtained following the clotting of blood samples at room temperature. The resulting samples were centrifuged at 1000× *g* for 10 min at 4 °C. The samples were kept on ice while handling. For lung samples, on day 14 post-challenge, mouse lungs were harvested, homogenized, and total IgG responses were determined in the supernatants, followed by centrifugation to remove any tissue debris. Briefly, 96-well plates were treated with purified RSV-F protein or inactivated virus (FI-RSV) at a concentration of 200 ng per well in coating buffer at 4 °C overnight. The plates were washed 3× with 0.1% Tween^®^20 in phosphate-buffered saline (PBST), then blocked with blocking buffer for 2.5 h at 37 °C. Plates were washed again with PBST. Serum or lung supernatants samples were diluted in 1× PBS at a 1:10 dilution for a total of 10 dilutions, added to the plates, and incubated at 37 °C for 2 h. Following another plate washing step, horseradish peroxidase (HRP)-conjugated goat anti-mouse IgG secondary antibodies (1:2000 dilution in PBST) were added and further incubated for 1.5 h. The substrate 3, 3′, 5, 5′-tetramethylbenzidine (TMB, BioRad) was used at room temperature for 3 min, and 0.16 M sulfuric acid was used as a stop solution. The optical density (OD) of the samples was then read at 450 nm using a BioTek plate reader. The cut-off value (COV) for the OD was established from serum or lung supernatants of control mice as previously described [11]. For each sample, the highest dilution with an OD value above the COV was equated to a positive titer [11]. For each group, the antibody titers were averaged to give the geometric mean titer and compared between weeks [11].

### 2.7. Lung Histopathology

Following euthanization, intact lungs were removed, following which, the left lobe was used for histological analysis, while the right lobe was homogenized for the determination of lung viral titers. Using a 3 mL syringe with a 22-gauge needle, the left lobe was slowly perfused with 1× PBS until it changed color. Lung tissues harvested from mice were fixed in 10% (*v/v*) formalin in 1× PBS for 24 h, washed with 70% ethanol, and embedded in TissueTek^®^ O.C.T. Compound. The specimens were stored frozen at −70 °C until further use. Sections of 10 μm were made using the cryostat microtome and collected on positively charged microscope slides. The slides were processed further for hematoxylin and eosin (H&E) staining to assess lung inflammation and necrosis post-challenge. Briefly, frozen slides were brought to room temperature and incubated with hematoxylin solution in a staining jar for 10 min to stain the nuclei. The slides were dipped a few times in a jar containing DI water to clean off any unused stain. The slides were submerged in the eosin Y stain solution for 3 min. Subsequently, the sections were transferred into staining containers with 70% ethanol for 20 s, 90% ethanol for 20 s, 100% ethanol for 1 min, and xylene for 3 min, as per a previously optimized protocol [19]. The slides were allowed to air-dry in a fume hood. Finally, the slides were mounted and sealed with coverslips [19]. Hematoxylin-and-eosin-stained images were taken using a digital microscope.

### 2.8. Lung Viral RNA Levels Determined with Real-Time PCR

The lungs from each group were harvested and homogenized with a cell strainer at day 6 post-challenge, and stored at −20 °C until further use. The procedure was performed as described previously by Chirkova et al. [20]. Total RNA was extracted from supernatants by using the Qiagen Total RNA extraction kit (Qiagen, Valencia, CA, USA) and stored at −80 °C. Real-time PCR was implemented with an AgPath-ID RT-PCR kit and an ABI 7700 sequence detection system [20]. Thermal cycling conditions and amplification cycles were optimized and followed [21]. The primers and probes for the RSV matrix (M) gene were used [21]. Threshold cycles (CTs) for each sample were calculated.

### 2.9. Statistical Analysis

The statistical analyses were performed using one-way analysis of variance (ANOVA) followed by Tukey’s multiple comparison test or two-way ANOVA in GraphPad Prism version 8 (version 8.3.1, GraphPad Software Inc., San Diego, CA, USA). A *p*-value less than 0.05 was considered significant. Error bars are indicative of the standard deviation of uncertainty.

## 3. Results

### 3.1. Characterization of Microparticles Encapsulating High Levels of RSV-F VLPs

**Figure 2:**
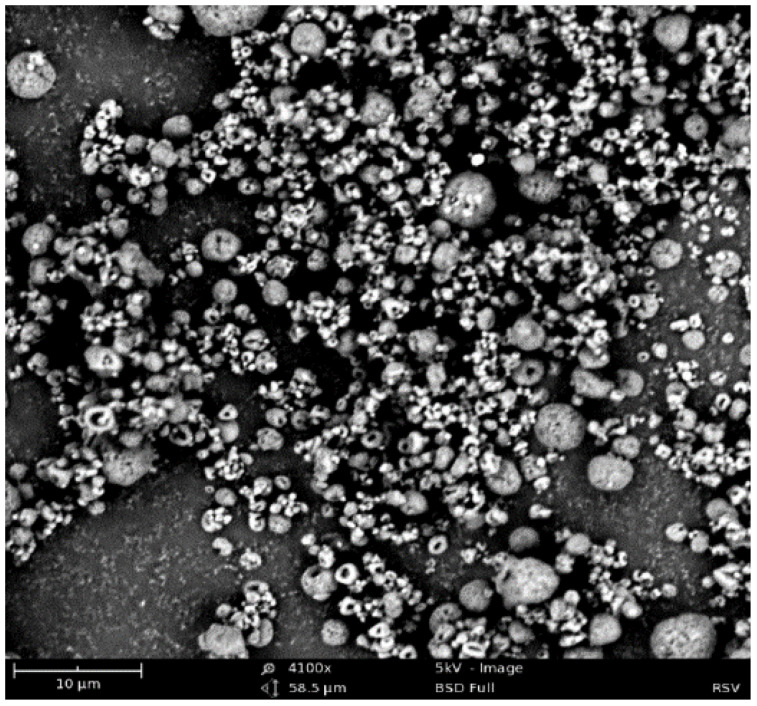
SEM images of RSV-F VLP-loaded microparticles. SEM images were captured using Phenom Desktop SEM^®^ by placing the microparticles on a conductive carbon tape and observed at 4100×, 5 kV. Microparticles exhibited an irregular shape with a particle size range of 1 to 4 µm.

The average percent yield of the MPs following spray drying was 88% *w/w*. The average size of the MPs was 2.53 ± 0.5 μm, with a size range of 1 to 4 μm. The microparticles were found to be positive with a charge of +25 ± 0.5 mV. The morphological characterization using SEM imaging showed irregularly shaped particles with surface indentations, as seen in Figure 2. The entrapment efficiency of the RSV-F antigen was approximately 85 ± 0.52%. The size range of the microparticles enables phagocytosis, and a positive charge allows for preferential uptake by dendritic cells.

### 3.2. Transdermal Vvaccination with RSV-F VLP + MPL^®^ Microparticles Induce IgG Antibodies

**Figure 3:**
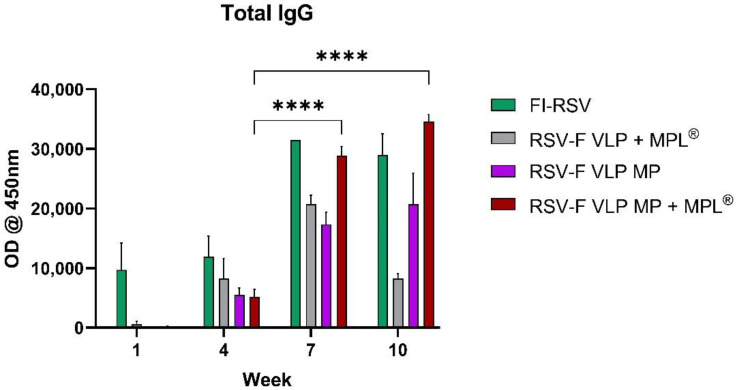
Total IgG measurement. RSV-F protein-specific antibody responses in mice. Mice received 1 prime and 2 booster doses of 5 µg/dose of F VLP transdermally and 1 µg/dose of FI-RSV (I.M.). The adjuvant group received 10 µg of MPL^®^. Serum samples collected at 1, 4, 7, and 10 weeks were analyzed for RSV-F protein-specific IgG using ELISA. Statistical significance was determined using two-way ANOVA in GraphPad Prism. Error bars represent standard error of the mean from individual animals. **** *p* < 0.0001.

Neutralizing antibodies are known to confer protection against respiratory viruses; hence, total IgG was quantified in serum. To determine the immunogenicity and efficacy of the RSV-F antigen, the groups of mice (*n* = 5) were immunized transdermally (T.D.) with RSV-F VLP MP, RSV-F VLP (5 μg) MP + MPL^®^ (10 μg) suspension or microparticles and I.M. immunized with FI-RSV (1 μg), and the control group was given 1× PBS (Figure 3). One prime dose was administered on week 1, followed by two booster doses administered at weeks 3 and 6, and serum was collected on weeks 1, 4, 7, and 10. RSV-F-specific IgG levels were measured in sera of mice vaccinated with microparticulate RSV F VLP using the purified RSV-F protein as the coating antigen (200 ng/well). RSV-F VLP MP + MPL^®^-treated immune sera showed significantly higher IgG titers than groups administered with RSV-F VLP + MPL^®^ suspension and non-adjuvanted RSV-F VLP MP on weeks 7 and 10, but not higher than the FI-RSV (I.M.) group (Figure 3). Antibody titers for the RSV-F VLP MP + MPL^®^ group showed a significant increase from week 4 to week 10 (Figure 2). The adjuvanted formulation, RSV-F VLP MP + MPL^®^, showed significantly higher IgG responses compared to the RSV-F VLP MP and RSV-F VLP + MPL^®^ suspension formulations (Figure 3). These results suggest that mice dosed with RSV-F VLP MP + MPL^®^ display significant levels of F-specific antibodies, thereby potentially enhancing the neutralization of the virus.

### 3.3. RSV-F VLP Microparticle Transdermal Vaccination Prevents Weight Loss

**Figure 4:**
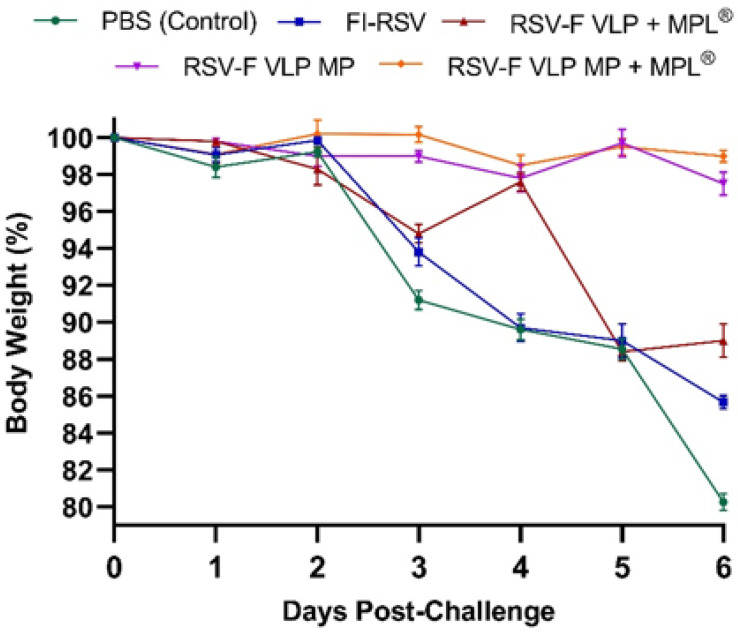
Body weight changes. Vaccinated mice (*n* = 5) were challenged with live RSV-A2 virus. The body weights were monitored daily, where 100% body weight was considered for day 0.

Mice treated with 1X PBS, FI-RSV, RSV-F VLP + MPL^®^ suspension, and RSV-F VLP MP with or without MPL^®^ were challenged with live RSV (1 × 10^6^ PFU) at 12 weeks following prime dose administration. The body weight of mice in all groups was monitored (Figure 4) to evaluate any signs of infection. A reduction in body weight was evaluated as a symptom of viral infection. The PBS-administered group showed >20% decrease in body weight, as did the FI-RSV (I.M.)-immunized mice (19%), compared to the F VLP MP + MPL^®^ group immunized via the transdermal route (<3%). Groups of mice that received RSV-F VLP MP and RSV-F VLP MP + MPL^®^ showed the least variation in weight after challenge. None of the groups that experienced weight loss were found to recover in the six days.

### 3.4. F VLP + MPL^®^ Microparticle Transdermal Vaccination Effectively Reduces Lung Viral Loads

**Figure 5:**
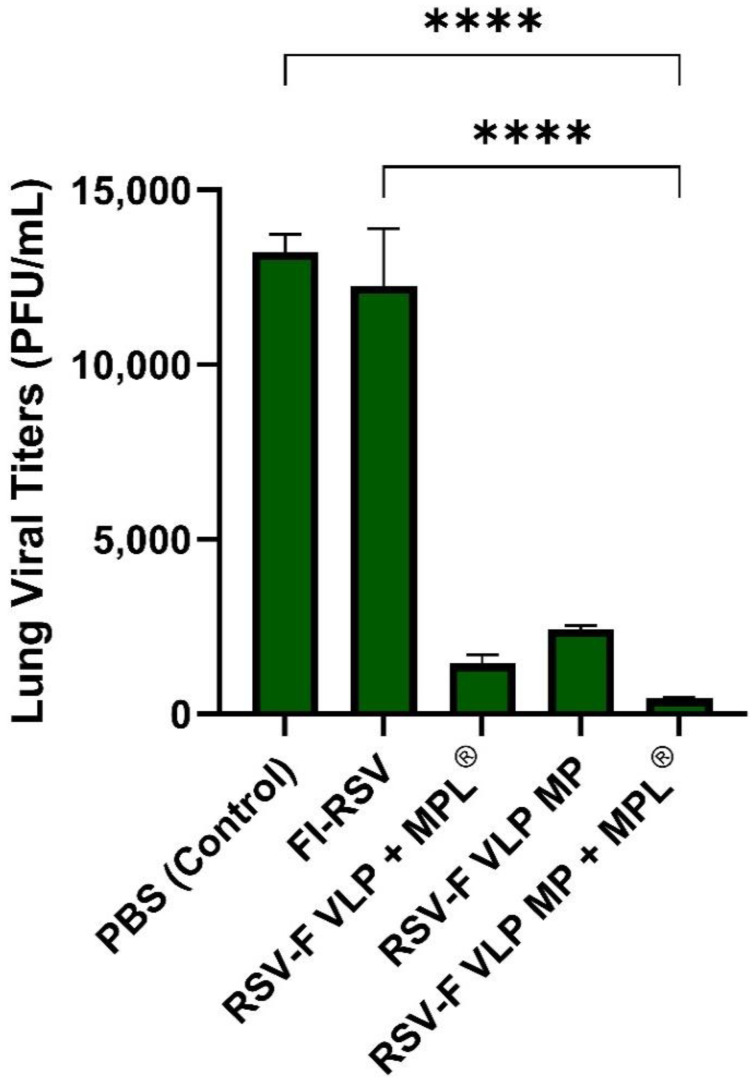
Lung viral titers quantified using RT-PCR. Lung samples from mice (*n* = 5) from all groups were harvested at week 14 and homogenized. Lung viral titers were quantified via extraction of total RNA and quantified using RT-PCR. Statistical significance was determined using one-way ANOVA in GraphPad Prism. **** *p* < 0.0001.

Lungs from mice (*n* = 5) were harvested on day 14 post-challenge and homogenized using a tissue homogenizer. The homogenized lung tissue was centrifuged at 3000× *g* for 10 min at 4 °C. Lung viral load was analyzed with a real-time reverse transcriptase-polymerase chain reaction (rRT-PCR) kit [20] to assess the protective efficacy of the vaccine formulations administered. It was observed that lung viral loads (PFU/mL) were cleared by 14 dpc (Figure 5). Decreased lung viral titers indicate an effective clearance of virus from the lungs and reduced inflammation, as evidenced by examining lung histopathology. The naïve group (PBS-treated) showed the highest lung viral load (1.6 × 10^4^ PFU/mL) at day 14 post-challenge, comparable to titers obtained from lung tissue of FI-RSV treated mice. Other groups that received treatments of F VLP antigens with or without adjuvants, and FI-RSV showed significantly lower viral titers, with RSV-F VLP + MPL^®^ exhibiting significant viral clearance, thus indicating a possible association with the production of serum-neutralizing antibodies. RSV-F VLP MP + MPL^®^ delivered via the Adminpatch^®^ microneedle array was most effective in lowering lung viral titers compared to all other groups (Figure 5).

### 3.5. Microparticulate RSV-F VLP Transdermal Vaccination Does Not Induce Pulmonary Histopathology upon RSV A2 Infection

**Figure 6:**
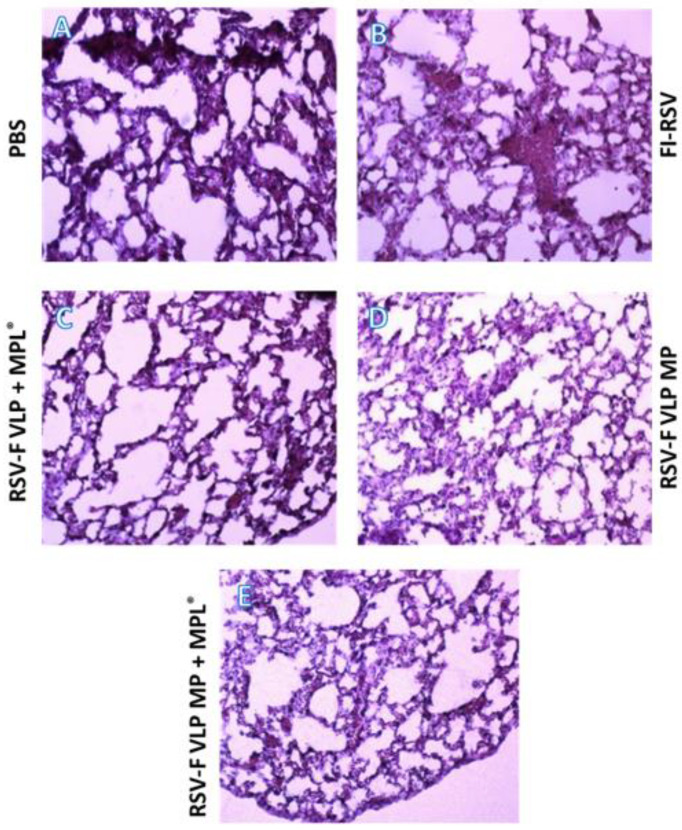
Lung tissues were collected from individual mice (*n* = 5 per group). Lung tissue sections were stained with hematoxylin and eosin (H&E) to assess inflammation. (**A**) PBS (control); (**B**) FI-RSV (I.M.); (**C**) RSV-F VLP + MPL^®^ (T.D.) suspension; (**D**) RSV-F VLP MP (T.D.); (**E**) RSV-F VLP MP + MPL^®^ (T.D.).

Lung histopathology is an essential parameter to evaluate the protective efficacy of an RSV vaccine. Significant inflammation was observed in the lungs of mice that received PBS (control group) (A.) and FI-RSV (I.M.) (B.) (Figure 6). The FI-RSV-administered group showed the highest degree of inflammation scores around the airways and interstitial spaces of the lung sections. The control group displayed moderate to substantial levels of lung histopathology following RSV infection. All other groups vaccinated with RSV-F VLP did not display evident inflammation, which illustrates that the F VLP antigen in particulate form proved to be a safe vaccine when given alone or with an adjuvant (MPL^®^).

### 3.6. F VLP + MPL^®^ Microparticle Transdermal Vaccination Induces F-Specific IgG Antibodies in Lungs Post-Challenge

**Figure 7:**
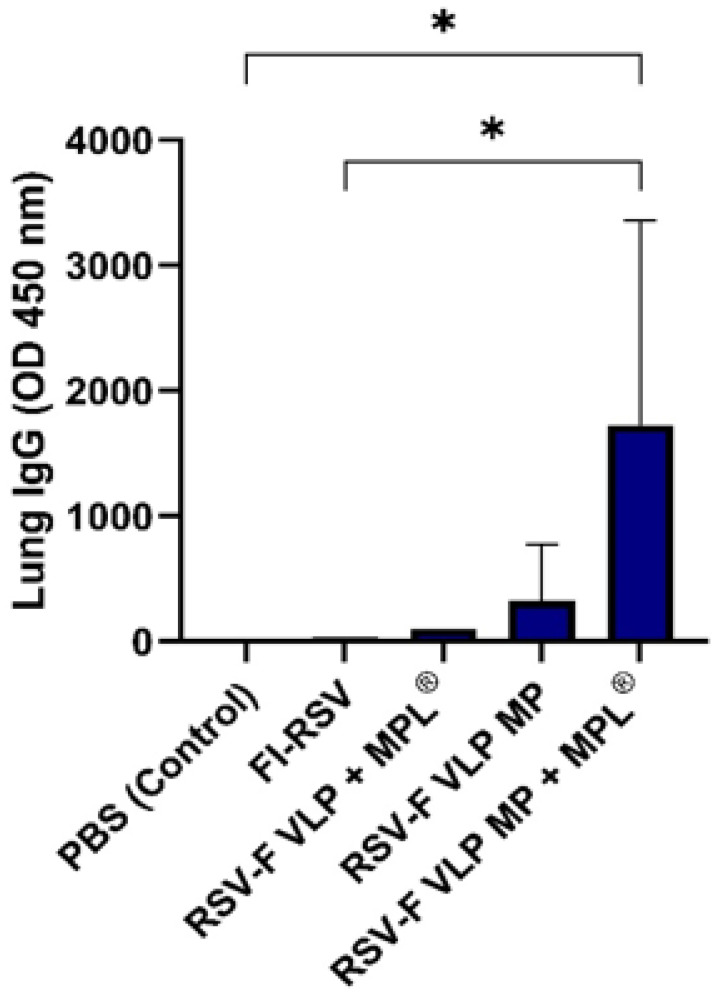
Measurement of lung IgG quantified using ELISA. Lungs were harvested and homogenized at week 14. The lung homogenates were centrifuged at 3000× *g*, and the supernatant was used for the assay. Statistical significance was determined using one-way ANOVA in GraphPad Prism. * *p* < 0.05.

At two weeks post-challenge, mouse lungs were collected and homogenized, and total IgG antibody responses were determined in the supernatants, following centrifugation to remove tissue debris. As shown in Figure 7, significantly higher levels of RSV-F-specific IgG antibodies were found in lung tissue from the mice immunized with RSV-F VLP MP + MPL^®^ (T.D.). These results indicate that the RSV-F VLP MP vaccine elicits RSV-F specific antibody responses in the lungs.

## 4. Discussion

Microparticulate delivery systems can stabilize vaccine antigens and ensure transport to intracellular compartments that improve vaccine immunogenicity, which subunit vaccines are unable to achieve on their own. Microparticulate vaccines, including the co-delivery of antigen and adjuvant, have been shown to allow targeted uptake by dendritic cells (DCs), which are central to innate and adaptive immune systems, thereby inducing strong humoral and cellular responses. In this study, a microparticle vaccine incorporating the RSV-F VLP antigen was spray-dried into a polymer matrix. Various immunological parameters in addition to viral load and lung histopathology were examined in mice following transdermal vaccination with RSV-F VLP in the presence or absence of an adjuvant such as MPL.

The microparticles prepared in this study were characterized for size, surface charge, surface morphology, and encapsulation efficiency. The spray-dried particles had a mean size of 2.5 µm, and previous studies have demonstrated that microparticles ranging from 1 to 5 microns elicit efficient immune-stimulating activity both in vitro and in vivo [11]. The encapsulation efficiency of RSV-F VLP in microparticles after spray drying was found to be 85%, which is considerably high for encapsulation efficiency in polymeric nano/microparticles as compared to other methods of preparation.

Developing an effective and safe RSV vaccine has posed a significant challenge, partially due to the exacerbated symptoms caused by the I.M. administration of FI-RSV [8]. Traditionally, most vaccines have been administered via the intramuscular (I.M.) route, as this route provides easy access to the appropriate immune cells required to produce a vaccine response. However, the fear of needles is still prevalent today. In recent times, the transdermal route has been widely researched for vaccines due to its patient acceptance and ease of administration, and especially because of the ability to benefit from the immunocompetence of the skin.

To investigate the efficacy of the transdermal route of administration, the RSV-F VLP microparticle vaccine was dosed in mice via the AdminPatch^®^ 1200. The data obtained in this study suggested that the efficacy of transdermal RSV-F VLP vaccines may be further improved with the addition of an adjuvant. The adjuvant used was a TLR-4 agonist, commonly referred to as MPL^®^, which was selected due to its widespread clinical and market acceptance. Per the study design, MPL^®^ was used in combination with VLPs since a previously published study demonstrated no protection in mice dosed with adjuvant alone, when compared to the group dosed with a combination of MPL and antigen [22].

To assess the immunogenicity of the RSV-F VLP microparticle vaccine, various immune correlates, including innate and adaptive immune responses, along with viral titers and lung histopathology were examined in mice. These robust immune responses are essential to eliminate the virus in the case of infection. With innate and adaptive immune pathways being distinct, the onset of adaptive immunity occurs after a longer period following infection, and hence, the evaluation of key immune parameters was carried out accordingly (at week 14 in this study). Additionally, RSV disease is known to progress to the chronic phase following day 14; therefore, to assess this, the mice were euthanized and immune organs along with the lungs were harvested to evaluate immune responses at that time point [23]. Strong immune responses capable of preventing infection and RSV disease progression following RSV vaccination remain poorly understood, limiting the approval of an RSV vaccine [7].

A key humoral response following vaccine administration is the increase in serum antibodies, particularly IgG. The F protein of RSV induces the production of neutralizing antibodies in the blood, offering enhanced protection from infection [5,8,9]. In this study, mice that received the RSV-F VLP MP + MPL^®^ vaccine showed significantly higher IgG titers compared to mice that received the suspension form or MP vaccine without adjuvant. The IgG titers were significantly elevated starting from week 4 to week 10. Although serum antibody levels or their ratios are a critical estimate, there is no defined approach to assess the extent of protection against RSV without taking into consideration other immune parameters [1]. In this study, the boost in total IgG levels in the group administered with RSV-F VLP MP + MPL^®^ coupled with the decrease in lung viral titers demonstrates the large dependence on RSV antibodies induced by vaccination to clear infection.

Similarly, in the lung, the measurement of antigen-specific antibodies is key to understanding RSV replication in the host and its spread to the bronchioles [24]. As seen in Figure 7, higher titers of RSV-F-specific IgG antibodies were found in lung homogenate supernatants collected from mice immunized with RSV-F VLP MP + MPL^®^ compared with FI-RSV and PBS controls, indicating a higher ability to neutralize the virus during infection, potentially inducing protection.

In murine models, body weight is seen as one of the major symptoms of RSV infection following challenge and is indicative of vaccine protective efficacy combined with viral load in the lungs [5]. Monitoring body weight changes following the live-virus challenge allowed us to determine if the mice developed immunity post-vaccination, which in turn would prevent viral infection. In this study, body weight changes were recorded for 6 days. This was based on previously published data with the RSV-F VLP vaccine, where the highest weight loss in certain groups of mice was observed by day 6 [5]. Insignificant body weight changes were observed in mice treated with the RSV-F VLP MP + MPL^®^. The FI-RSV and PBS control groups showed a significant decrease in body weight upon infection compared to all other groups.

Lung viral load is an important measure of the degree of infection, indicative of effective viral clearance, and correlates to RSV-specific antibody levels in the blood [7]. This immune parameter offers assurance that the immune response following vaccination is working against the spread of RSV disease. The RSV-F VLP MP + MPL^®^-immunized mice exhibited the lowest lung viral titers among all groups (Figure 5). Consistent with weight loss and low viral clearance, FI-RSV-vaccinated mice showed increased pulmonary inflammation when observed alongside the VLP-vaccinated groups (Figure 6). Transdermal immunization with RSV-F VLP MP + MPL^®^ prevented pulmonary inflammation following challenge with live RSV A2, similar to the RSV-F VLP MP and RSV-F VLP + MPL^®^ (suspension) groups (Figure 6). RSV-F VLP MP + MPL^®^ immunization did not produce pulmonary edema as observed in RSV disease and also resulted in significantly lower viral titers, as demonstrated in Figure 5 and Figure 6. The addition of a TLR-4 agonist such as MPL^®^ as an immune modulator may offer protection to the host from vaccine-enhanced pathology, and reduced RSV replication in the lower respiratory tract, as seen from the IgG titer and lung viral load results [24,25]. The results in this study suggest that mice immunized transdermally with F-VLP + MPL^®^ may induce protective immune responses, as inconsequential body weight changes and inflammation were observed following the challenge with live RSV-A2 virus. These results provide proof that a microparticulate RSV-F VLP + MPL^®^ vaccine administered transdermally significantly lowered lung inflammation and lung viral loads compared to IM injection.

In addition to humoral immunity, it is important to understand cellular immunity and the role it plays in the activation of the adaptive immune system. The two main effector T-cell populations, CD4^+^ and CD8^+^, play critical roles in eliciting protective immunity after antigen presentation and proceeding downstream events. Cellular responses evaluated in the lungs and lymphoid tissue (data not provided) are important to understand RSV pathology.

Future in vivo studies should warrant the evaluation of CD4^+^ and CD8^+^ T-cell counts in the lungs or bronchoalveolar lavage (BAL) fluid. Studies show that CD4^+^ memory T cells residing in the lung could be potential targets for boosting long-term cellular immunity following vaccination [26]. Hence, the evaluation of CD4^+^ and CD8^+^ T memory cells following vaccination would provide insight into long-term vaccine efficacy, making them an important population to induce through vaccination.

In summary, the transdermal RSV-F VLP vaccine induced robust immune responses and emphasized the benefit of the microparticulate delivery system for vaccine administration. Moreover, the results from this study form a basis for the future exploration of a potential novel RSV vaccine candidate.

## 5. Conclusions

Despite several years of research, most attempts at developing a safe and effective RSV vaccine have been unsuccessful, owing to various immunological factors. A potential vaccine formulation for the transdermal delivery of the RSV-F VLP antigen with or without MPL^®^ using a biodegradable polymer matrix was successfully developed and tested. The various immune correlates measured in this study provide insight into the development of a vaccine using a novel particulate delivery system in combination with an adjuvant, enhancing its efficacy when delivered transdermally. Thus, this study demonstrated the ability of an adjuvanted RSV-F VLP microparticulate vaccine to induce a robust response against RSV infections.

## Figures and Tables

**Table 1:** Groups for animal study. Mice (*n* = 5, per group) were dosed with RSV-F VLP with or without adjuvant. The control group of mice was administered PBS and served as the negative control, while the formalin-inactivated RSV (FI-RSV) administered group was selected as a positive control.

Groups	Antigen Dose (µg)	Route of Administration	No. of Mice
Control (1× Phosphate-Buffered Saline)	N/A	T.D.	5
Formalin-inactivated RSV (FI-RSV)	1	I.M.	5
RSV-F VLP + MPL^®^ (Suspension)	5	T.D.	5
RSV-F VLP (MP)	5	T.D.	5
RSV-F VLP + MPL^®^ (MP)	5	T.D.	5

T.D. = transdermal; I.M. = intramuscular; MP = microparticle. N/A = not applicable.

## Data Availability

Data will be made available upon reasonable request.
